# Influence of Periodontitis, Implant, and Prosthesis Characteristics on the Peri-Implant Status: A Cross-Sectional Study

**DOI:** 10.1155/2022/9984871

**Published:** 2022-02-07

**Authors:** Ioanna Papalou, Panagiota Vagia, Ahmet Cakir, Henri Tenenbaum, Olivier Huck, Jean-Luc Davideau

**Affiliations:** ^1^Department of Periodontology, Dental Faculty, University of Strasbourg, 8 Rue Sainte-Elisabeth, Strasbourg 67000, France; ^2^Pôle de Médecine et Chirurgie Bucco-Dentaires, Hôpitaux Universitaires de Strasbourg, Strasbourg 67000, France

## Abstract

**Background:**

The association between peri-implant diseases and the periodontal, implant, and prosthesis characteristics has been characterized in various ways.

**Purpose:**

The aim of this study was to evaluate the link between the peri-implant and periodontal status and the influence of implant and prosthesis parameters during implant follow-up.

**Materials and Methods:**

One hundred and seven patients with a total of 310 implants that had at least one year of function who were attending periodontal and implant maintenance at a university clinic setting were included in this cross-sectional study. The demographic, periodontal, peri-implant tissue, implant, and prosthesis parameters were recorded. A pocket depth > 4 mm with bleeding on probing defined periodontal/peri-implant soft tissue diseased sites. Analyses were performed at the patient and implant levels using univariable and multivariable mixed regression analysis.

**Results:**

The mean implant follow-up was 7.22 years. At the patient level, the bleeding on probing and pocket depth measurements were more pronounced around the implant than around the teeth. The opposite was observed for plaque and the clinical attachment levels. At the implant level, multivariable analysis showed that the periodontal and corresponding peri-implant tissue parameters, such as diseased sites, were closely related. The implant location, bone level, and number were selectively associated with the implant bone level, while cemented retention and emergence restoration profile influenced the implant pocket depth.

**Conclusions:**

The present study suggested that clinical peri-implant and periodontal soft tissue statuses were different, which could be a consequence of the initial implant and prosthesis healing process. However, during implant follow-up, the peri-implant parameters were predominantly associated with their corresponding periodontal parameters regardless of an association with the implant and prosthesis characteristics. This trial is registered with ClinicalTrials.gov ID: NCT03841656.

## 1. Introduction

Peri-implant diseases are mainly characterized by the inflammation of peri-implant tissues and the progressive loss of supporting bone around implants potentially leading to implant failure [1, 2]. Bone loss could be due to peri-implant mucosa infection that corresponds to the definition of peri-implantitis [3] and/or to immune response that corresponds to the definition of a foreign body reaction [2]. The diagnosis of peri-implantitis is mainly based on various clinical parameters reflecting abnormal inflammation and destruction around implants, such as bleeding on probing (BOPi) and/or suppuration, an increase in peri-implant probing depth (PiPD), and radiographic evidence of bone loss that has occurred after the initial healing [4]. However, definitions of peri-implantitis varied greatly between studies, which was based on the various combinations of clinical signs with various levels of severities [1, 5] and led to the peri-implantitis prevalence being very variable and ranging from 1 to 47% [6]. Recently, the 2017 World Workshop on the Classification of Periodontal and Peri-Implant Diseases and Conditions proposed a definition of peri-implantitis [4]. This definition is based on the combination of BOPi/suppuration presence, the longitudinal assessment of PiPD and bone level changes; or as an alternative, it was based on specified thresholds, i.e., PiPD ≥ 6 mm and bone level ≥ 3 mm at least in one site around the implant. However, correlations between different peri-implant tissue characteristics, such as the mean PiPD, BOPi, and bone level (BLi), as well as between these parameters and peri-implantitis did not systematically correspond [7], which was contrary to the classically observed periodontal parameter associations in periodontal diseases [8] and during periodontal maintenance [9]. Various peri-implant tissue parameters that are used to define disease severity and activity/progression may not only correspond to the responses of the host's peri-implant tissues to plaque/biofilm accumulation or implant foreign body but also reflect the complex and specific influence of the periodontal environment and implant/prosthesis procedures [2, 3, 7, 10].

The strength of the association between periodontal and peri-implant diseases varies greatly depending on the disease definition and assessment times [1, 5]. There is high heterogeneity in cross-sectional studies evaluating the link between the present periodontal status and peri-implantitis/bone loss [11]. Their results showed that disease parameter selection impacted the relationship between periodontal and peri-implant diseases. For instance, cut-off values of 5% periodontal pocket probing depth (PPD) ≥ 4 mm and bleeding on probing around teeth (BOP) ≥30% per patient were associated with the mean and percentage of PiPD ≥4 mm and BOPi but not with the mean bone level around the implant (BLi) [12]. In the study of Pjetursson et al. [13], the mean PPD and residual pockets with % PPD ≥5 mm were associated with two different definitions of peri-implantitis based on the presence of BOPi, BLi ≥2 mm, and PiPD ≥5 or 6 mm around implants. BOP was only associated with the first one (PiPD ≥5 mm) and the mean periodontal clinical attachment level (CAL) was only associated with the second one (PiPD ≥6 mm) [13]. As each periodontal parameter and combination represented different aspects of periodontal disease pathogenesis/morbidity, severity, complexity, treatment response, and progression [14], these different associations between the clinical parameters suggested that the characteristics/profile of patient responses to periodontal treatment may selectively impact the peri-implant status.

The surgical and prosthesis procedures, as well as the type of implant, have been shown to impact the peri-implant conditions such as PiPD and BLi around implants as well as their follow-up changes [1, 4, 15–18]. For instance, both the anterior-maxilla implant location and bone-level implant design were associated with an increased bone loss after implant healing [16]. Reduced keratinized mucosa conditions were associated with more plaque accumulation and inflammation around the implants [19]. PiPD was increased in the case of cemented retention prosthesis [20]. The prosthesis contour and type were inconsistently associated with peri-implantitis [17, 18, 21–24]. The combination of prosthesis factors, such as an emergence angle >30 degrees and bone-level implants, could amplify the risk of peri-implantitis [18, 22]. However, the respective influence of implant/prosthesis factors and the periodontal status on the peri-implant status was not clearly established [21].

The difference between periodontal and peri-implant tissue histology, physiology, and pathogenesis could influence the diagnostic relevance of related clinical signs and consequently the evaluation of risk factor impacts [3, 7, 25]. The variability of peri-implant tissue conditions after implant and prosthesis healing did not allow for a definition of pre-established probing depth, attachment, and bone level with normal and pathologic values [25]. Peri-implant tissue parameter changes are now allowed for diagnosing peri-implantitis and the influence of risk factors [26]. However, beyond definitions of health and disease states, there is also a need to evaluate the link between periodontal and peri-implant tissue conditions during the implant follow-up. The purpose of the present cross-sectional study was to evaluate various clinical parameters defining the peri-implant status and the periodontal, implant, and prosthesis status in patients attending supporting periodontal and implant therapy and to investigate the associations between the parameters.

## 2. Materials and Methods

### 2.1. Study Population

The Ethical Committee of Strasbourg University Hospital has independently reviewed and approved this cross-sectional study (AMK/BG/2016-95 – ClinicalTrials.gov- https://ClinicalTrials.gov ID: NCT03841656). The subjects gave their written consent to participate after being informed of the study objectives. The study was conducted according to the principles stated in the Declaration of Helsinki [27]. To be included in this study, participants had to fulfill the following criteria: (a) patients had one or more implants for at least 1 year of function that was placed at the Department of Periodontology of the Dental Faculty, University of Strasbourg, (b) patients had the initial periodontal diagnosis and the active and supporting periodontal therapy performed at the same Department of Periodontology before the implant placement, (c) patients had available and reliable updated demographic, medical, periodontal, and implant data, (d) patients were ≥20 years at implant placement, and (e) patients had at least 12 residual teeth at the implant placement. Patients in need of antibiotic prophylaxis for clinical periodontal examination and treatments were excluded.

Dentate adults who had undergone periodontal and implant treatment from 1999 to 2017 at the Department of Periodontology of the Dental Faculty of Strasbourg were identified from the clinic's database as previously described [28]. Initial periodontal diagnosis, i.e., gingivitis/mild periodontitis, moderate periodontitis, and severe periodontitis based on criteria defined by the 1999 International Workshop for a Classification of Periodontal Diseases and Conditions [29], was established from patient file data. After active periodontal therapy (APT) was completed, the supporting periodontal therapy (SPT), scaling and root planing were performed in residual and recurrent sites with PPD ≥4 mm. Periodontal surgery was performed during APT and SPT in cases of persistence or recurrent sites with PPD ≥6 mm. The frequency of SPT sessions ranged from 3 to 6 months depending on the APT and SPT outcomes. During periodontal follow-up, 310 implants (Straumann, AG®, Basel, Switzerland) were placed to replace missing teeth at the end of APT and during SPT. After implant placement, some patients were referred to their private practitioners for maintenance care. At every recall visit/examination, all-evident pathologic peri-implant conditions were recorded and treated according to implant maintenance and treatment protocol (Cumulative Interceptive Supportive Therapy–CIST) [30].

**Figure 1:**
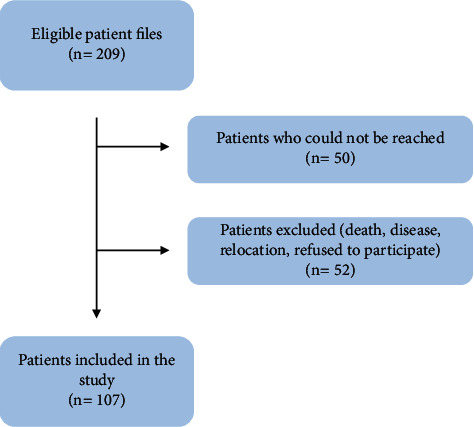
Study flowchart.

After screening all completed files, 209 patients who met the inclusion criteria were contacted for a clinical examination between September 2017 and December 2019. Among them, 50 patients could not be reached, while 52 reachable patients or their families were excluded either due to death, difficulty in attending the recall appointment (disease, relocation), or refusal to participate in the study for various personal reasons, such as ethical reasons or dissatisfaction. Finally, 107 patients were available for a clinical and radiographic final re-examination (Figure 1).

### 2.2. Examination

Periodontal and implant examinations were performed at the final re-evaluation. Demographic data, medical history, and smoking status data were recorded. Regarding smoking, patients were divided into 3 groups: the nonsmokers (who had never smoked), former smokers (who quit > 5 years ago), and current smokers (who had at least one cigarette/day). At the re-evaluation, the periodontal status of patients was classified according to the 2017 World Workshop on the Classification of Periodontal and Peri-implant Diseases and Conditions [14]. Clinical examination was performed by two calibrated examiners (IP and PV). The full-mouth plaque score (FMPS), PPD, CAL, gingival recession (REC), and BOP around teeth were recorded. The PiPD, clinical attachment level (CALi), mucosa recession (RECi), implant plaque score (IPS), BOPi, and suppuration around implants were recorded. All measurements were performed manually at six aspects of each tooth and implant using a PCPUNC 15 probe (HuFriedy, Chicago, IL, USA). For CALi, the implant platform/shoulder was considered the cervical limit [21, 31].

### 2.3. Implant and Prosthesis Characteristics

Tissue-level (*n*=295) and bone-level (*n*=15) implants were placed in different locations, including the anterior-maxilla, anterior-mandible, posterior-maxilla, and posterior-mandible. The width of keratinized mucosa was measured using a PCPUNC 15 probe at buccal sites [32]. The emergence angle of the implant prosthesis was defined as the angle between the tangent of the prosthesis contour relative to the implant long axis as previously described [18, 22]. The prosthesis retention type, cement or screw retention, prosthesis type, bridge, and single crown were recorded [21, 24, 33]. Misfitting was defined by radiographic evidence of an open margin between the abutment and restoration [17].

### 2.4. Radiographic Analysis

A radiographic examination was performed with digital orthopantomography and periapical radiographs (Planmeca, Roselle, IL, USA) obtained using the long-cone parallel technique and Rinn system (XCP Instruments, Rinn Corporation, Elgin, IL, USA). Measurements were performed with Centricity Enterprise web-specific software (GE Medical Systems IT, Wauwatosa, WI, USA). Bone level around the implant was measured on periapical radiographs as the distance from the junction between smooth and rough implant surfaces for the tissue-level implants and from implant shoulder for bone-level implants to the first bone-to-implant contact on mesial and distal aspects of the implants by the same two calibrated examiners (I.P. and P.V.). The most elevated measurement (mesial or distal) was selected to represent BLi [33]. Estimation of bone loss in relation to patient age (BL/age) was performed on orthopantomography for the site that was affected the worst [14].

### 2.5. Case Definition for Patients with Peri-Implant Mucositis and Peri-Implantitis

Peri-implant mucositis was defined as the presence of BOPi without PPD ≥6 mm and a bone level ≥3 mm apical to the most coronal portion of the intraosseous part of the implant. Peri-implantitis was defined as the presence of PiPD ≥6 mm with BOP or suppuration and radiographic signs of a bone level ≥3 mm apical to the most coronal portion of the intraosseous part of the implant, according to the case definition of Berglundh et al. [26]. This definition was used in recent cohort studies [20, 34]. The other cases were considered peri-implant health.

### 2.6. Examiner Calibration

Examiners underwent inter-examiner calibration on patients not included in the study. The percentages of agreement between the two examiners (I.P. and P.V.) within ±1 mm for probing depth and attachment level and within ±0.5 mm for BLi were 96.3%, 79.6%, and 83.78%, respectively. The intraclass correlation coefficients were >0.8.

### 2.7. Statistical Analysis

At the patient level, periodontal and corresponding peri-implant characteristics were compared using the Wilcoxon test. For the analysis at implant level, implants at anterior mandible and anterior maxilla locations were grouped into a single group due to the low number of anterior mandibular implants. Considering that more than one implant could be placed in each patient, mixed models were used to analyze the associations between demographic, periodontal, implant, prosthesis characteristics, and peri-implant parameters. The patients were used in these models as random effects [34]. Multivariable regression analysis was performed for each peri-implant tissue parameter with demographic, treatment, implant, and prosthesis parameters that presented *P* < 0.2 in the univariable regression analysis and the corresponding periodontal parameters. Differences were considered significant with *P* < 0.05. Analyses were performed using statistical software (XLSTAT, Addinsoft, Paris, France).

## 3. Results

### 3.1. Demographic Characteristics of the Studied Population

The ratio between the finally included and eligible patients was 51%. One hundred and seven patients with 310 implants that had functioned for at least one year were included in the study. The mean patient age was 66.2 years, and the percentage of women was 54.2%. The percentage of current smokers was 11.21%, and most of them (10/11) did not smoke more than 10 cig/day. Six patients (5.6%) had stabilized diabetes.

### 3.2. Treatment, and Periodontal and Peri-Implant Characteristics at the Patient Level

At the initial visit, 90 (84.11%) of the patients suffered from moderate and severe periodontitis. At re-evaluation, 2 (1.86%), 38 (35.51%), and 67 (62.62%) patients were diagnosed with stage I, stage II, and stage III/IV periodontitis, respectively. In the studied population, 90 (84.11%) and 3 (2.80%) patients suffered from peri-implant mucositis and peri-implantitis, respectively. The mean percentage of sites with both PiPD ≥6 mm and BOPi was 0.75 ± 2.96. The number of patients with at least one implant with a BLi ≥3 mm was 11 (10.24%). During implant follow-up, three implants were lost for peri-implantitis reason, and eight implants were lost for the absence/lost of osteointegration or implant fracture reasons. The total mean patient follow-up was 12.82 years. The mean age of implant surgery per patient was 58.35 years (±9.3). The mean time as a function of implants per patient was 7.22 years, ranging from 1 to 17.95 years. There were 74 (69.15%) patients with a mean time in function >5 years. The mean numbers of teeth and implants were 21.91 and 2.95, respectively, and 33 (30.84%) of the patients had at least three implants placed. Twenty-four (22.4%) patients had implants placed at different times (interval >2 years). The comparison between periodontal and peri-implant characteristics showed that plaque accumulation was more prevalent around teeth (FMPS = 23.64) than around implants (IPS = 16.5). Conversely, the percentage of BOP was more pronounced around implants (BOPi = 25.91) than around teeth (BOP = 14.4). The mean PiPD (2.72 mm) was higher than the mean PPD (2.4 mm). The % of sites with both PiPD >4 mm and BOPi (PiPD >4 mm + BOPi) was 4.77 and was higher than % PPD >4 mm + BOP (1.58). The mean CALi (2.73 mm) and % RECi >1 mm (4.21) were lower than the mean CAL (3.29 mm) and % REC >1 mm (27.59). The BL/age was 0.58 mm, while the BLi around implants was 0.86 mm (Table 1).

### 3.3. Associations between the Demographic, Periodontal, and Peri-Implant Tissue Characteristics at the Implant Level Using Univariable Regression Analysis

Few associations were observed between the demographic, smoking, diabetes, and peri-implant tissue parameters. The patient age was associated with a reduction in BOPi. The number of implants per patient was associated with increases in the mean CALi and BLi values and BOPi decreases. Women had more % PiPD >4 mm + BOPi than men. Among the periodontal characteristics, FMPS was significantly associated with IPS, mean PiPD, and CALi increases, and was nearly significantly associated with BLi (*P*=0.059) and BOPi (*P*=0.069). BOP was only associated to BOPi. The mean PPD was associated with the IPS, mean PiPD, and CALi. % PPD >4 mm + BOP was associated with the mean PiPD, CALi, and % PiPD >4 mm + BOPi. The mean CAL was associated with IPS, mean PiPD and CALi, and % PiPD >4 mm + BOPi. No significant association was observed for the BL/age (Table 2).

### 3.4. Implant and Prosthesis Characteristics

As observed at the patient level, the percentage of implants with peri-implantitis was low (0.96%). Twelve (3.87%) implants had a BLi ≥3 mm. The mean time in function per implant was 7.73 years. The majority of implants were placed in the posterior location, with similar percentages at the maxilla (40.65%) and mandible (41.61%). At the anterior location, 16.45% of the implants were placed at the maxilla, while only 4 implants were placed at the mandible. The tissue-level implants represented 95.16% of the implants. The mean width of keratinized mucosa was ≥2 mm for 73.55% of the implants. More than 90% (91.21%) of the restorations were cemented. The distal and/or mesial emergence profile angle was >30 degrees for 38.06% of the implant restoration contours and 38.06% of the implant-supported bridge restorations. Finally, 19.68% of the prostheses displayed misfitting (Table 3).

### 3.5. Association between the Implant, Prosthesis, and Peri-Implant Tissue Characteristics at the Implant Level Using Univariable Regression Analysis

Time as a function of implant was only associated with a decrease in BOPi. The implant location was associated with the mean PiPD, % PiPD >4 mm + BOPi, and Bli. Implants in the posterior mandible location exhibited lower mean PiPD and % PiPD + BOPi >4 mm values than implants in the posterior maxilla location. Implants in the anterior location had more pronounced Bli than the implants in the posterior maxilla location. The bone-level implants were associated with a higher Bli than the tissue-level implants.

Among the prosthesis characteristics, a thick peri-implant mucosa condition was associated with BOPi and a mean PiPD increase. Cemented prostheses were associated with higher mean PiPD and CALi. An emergence profile angle >30 degrees was associated with a lower mean PiPD and mean CALi. Bridge restoration was associated with higher Bli and lower BOPi, while misfitting was associated with higher mean PiPD and lower IPS (Table 4).

### 3.6. Independent Association between the Periodontal, Implant, and Prosthesis Characteristics and Peri-Implant Parameters at the Implant Level Using Multivariable Regression Analysis

Multivariable regression analyses showed that the mean CAL and FMPS values were only independently associated with the corresponding mean CALi and IPS values. These analyses confirmed that the mean PPD, BOP, and % PPD >4 mm + BOP values were associated with their corresponding peri-implant parameters. There was a strong independent association between the BL/age and Bli. There were few persisting associations between the peri-implant parameters and demographic, implant, and prosthesis characteristics. For instance, BOPi was reduced in smokers. The Bli appeared to be most influenced by the implant characteristics, while the mean PiPD appeared most influenced by the prosthesis characteristics. The % PiPD >4 mm + BOPi was similarly influenced by gender, implant, and prosthesis parameters (Table 5).

## 4. Discussion

Peri-implant diseases are classically associated with periodontal diseases but are less consistently dependent on implant and prosthesis factors [1, 5]. The strength of these associations greatly depends on the peri-implant morbidity definitions, risk factor definitions, and combinations of the two. The present cross-sectional study in patients having periodontal and implant maintenance demonstrated that the periodontal and peri-implant statuses were associated during follow-up regardless of the impacts of implant and prosthesis procedures.

The total mean time of periodontal and implant follow-up was 12.82 years. At re-evaluation, almost all patients presented with at least stage II periodontitis as defined by the 2017 World Workshop on the Classification of Periodontal and Peri-Implant Diseases and Conditions [14], and 62.62% presented with stage III or IV periodontitis, confirming the periodontal risk of the studied population. The mean time of function for implants was 7.22 years, and almost 70% of patients had a mean time >5 years, which shows the long-term nature of implant follow-up. The percentage of peri-implantitis per patient was 2.80%, and it was considerably lower than in other studies (24.8% [18] and 41.4% [20]) with similar peri-implantitis definitions and mean implant follow-up durations. This low prevalence could be explained by the low percentage of behavioral and systemic risk factors, such as smoking ≥10 cig/day, inadequate plaque control, and the diabetes that was observed in the present study, contrary to the results described by Kissa et al. [20]. Furthermore, this low prevalence could also be due to the efficiency of CIST performed during implant follow-up, which could reduce the prevalence of peri-implantitis at re-evaluation. For this reason, implants previously treated for peri-implantitis have not been considered at the final examination in the study of Yi et al. [18]. Interestingly, 11 (10.28%) of the patients presented at least an implant with a Bli ≥ 3 mm. The possibility that bone loss around implant may not involve peri-implant mucosal infection and inflammation similarly to periodontitis but corresponds more to an abnormal bone immune response to a foreign body is still debated [1, 2, 25]. However, many studies have shown that periodontal and implant diseases were associated [1, 5], suggesting the existence of shared physio-pathological mechanisms and/or risk factors [3].

The peri-implant tissue measurements, mean PiPD (2.72 mm), BOPi (25.9%), and Bli (0.86 mm) appeared lower than the overall mean weighted PiPD (3.3 mm), BOPi (52.2%), and bone loss (1.1 mm) described in a recent review [7], confirming that the studied population had good peri-implant tissue conditions. At the patient level, periodontal and peri-implant soft tissues displayed significantly different parameter distributions. Plaque accumulation was less pronounced around the implants than around the teeth, while in other studies, the plaque scores were similar [31, 35]. The lower IPS could be due to a lower adhesion of biofilm to implant and prosthesis surfaces, as observed during experimental gingivitis/mucositis [36]. The BOPi and mean PiPD were higher than the BOP and mean PPD. Such differences have been previously described in studies comparing implants to their matching control teeth [31] and implant probing to full mouth tooth probing [35]. The higher BOPi could be due to a reduced resilience of peri-implant tissue to probing [37] or a higher proinflammatory state, while the higher PiPD could be due to specific soft tissue histologic characteristics and healing around the implant [7, 38]. The % PiPD >4 mm + BOPi was also higher than % PPD >4 mm + BOP, in accordance with mean PiPD/PPD and BOPi/BOP, observed differences. The mean CALi and % REC >1 mm values were less pronounced than in the corresponding periodontal parameters, as previously observed for the mean CALi and RECi in a study using control teeth [31]. Peri-implant mucosa recessions were mainly considered as the consequences of implant procedures, the lack of buccal bone and keratinized tissues, and surgical treatments [1]. However, CALi and RECi longitudinal changes could also be observed in the long term [31]. These data suggested that peri-implant status could reflect the initial influence of the implant healing process. However, pronounced changes in peri-implant tissue parameters have been more or less observed after healing during a ten-year follow-up, highlighting a potential adaptation to their environment [31].

At the implant level, both univariable and multivariable mixed regression analyses demonstrated that there were only a few associations between the age, gender, smoking, and peri-implant tissue parameters. A significant increase of % PiPD >4 mm + BOPi was only observed in women. Such a BOPi increase in women has been previously observed and was suspected to be due to hormonal influence [39]. However, the impact of gender on peri-implant status is not frequently observed [5]. The smoking status did not negatively impact the peri-implant parameters, while it was considered a major risk factor for peri-implant and periodontal diseases [1]. The low percentage of smokers and their moderate cigarette consumption of mainly less than 10 cig/day may explain the limited effect of smoking [40]. Similarly, the impact of the follow-up time on Bli appeared limited, as shown in previous studies using similar types of implants [16, 41]. The low impact of time was described for the occurrence of peri-implantitis in some studies [42, 43]. Conversely, univariable regression analyses showed that the number of implants per patient appeared to be more associated with the peri-implant conditions. The impact of implant number on peri-implant conditions has been previously observed for peri-implantitis in some studies [23, 44, 45]. The number of implants was correlated with tooth loss, which could reflect the impact of periodontal risk indicators [24], as suggested for implant failure [46]. Using multivariable analysis, the impact of implant number on the peri-implant tissue parameters was reduced, as shown for peri-implantitis [31, 47]. However, it was still present for Bli, suggesting that other risk factors specifically correlated with the implant number may influence peri-implant soft tissue conditions [23].

Periodontal and peri-implant conditions were clearly associated, but the strength of associations greatly varied depending on the selected parameters. Univariable regression analyses indicated that the patient plaque score appeared to be thoroughly associated with the peri-implant condition parameters, confirming the observed overall impact of patient oral hygiene efficiency on peri-implant conditions [1]. The mean PPD was associated with various aspects of peri-implant tissue conditions, such as IPS, PiPD, and CALi, suggesting that mean PPD could strongly reflect the influence of periodontal status [31]. For other periodontal characteristics, the number of associations appeared more limited. Parameters related to periodontal tissue destruction, such as the mean CAL and BL/age, were mainly associated with their corresponding peri-implant characteristics. Periodontal inflammation/BOP was only associated with BOPi. However, the impact of BOP alone on peri-implant conditions, such as peri-implantitis, has been previously observed [43, 48] but not systematically observed [13, 42]. The % PPD >4 mm + BOP was associated with % PiPD >4 mm + BOPi increase. The absence of residual sites with both PPD >4 mm and BOP was defined as the endpoint of a successful periodontal therapy [49]. Their persistence during SPT may signal periodontitis persistence/activity [50]. Similarly, PiPD >4 mm + BOPi could be considered as a clinical sign of peri-implant disease associated with the implant bone loss [51]. These patterns in the associations suggested the existence of exclusive clinical relationships between the various components of periodontal and peri-implant disease diagnosis.

The implant and prosthesis characteristics impacted the peri-implant conditions less than periodontal characteristics did. As observed for the periodontal parameters, univariable regression analysis showed that there were specific patterns of associations with peri-implant parameters. Both the PiPD and Bli were notably influenced by the implant location. Implants placed in the posterior mandible displayed less PiPD than those in other locations, as previously demonstrated for PiPD and/or Bli [16, 31]. Less impaired peri-implant conditions at the mandible have also been described for peri-implantitis [17] but not in a systematic manner [5, 52]. The Bli increase was associated with bone-level implants, as previously observed [16, 18], and could be due in part to initial bone remodeling [16]. The mean PiPD increase was associated with cemented prostheses, suggesting a potential irritation of the cement remnants [1]. The impact of the prosthesis characteristics appeared limited. Bridge restoration was associated with an increase in Bli, as previously observed [18]. This effect could be related to mechanical overloading [24] and/or limited accessibility for oral hygiene [18]. However, bridge restorations and factors impacting the shape of interproximal spaces, such as the emergence profile angle, were not associated with IPS increases in the present study. Interestingly, a decrease in BOPi was also associated with bridge restorations. The use of interproximal brushing could compensate for the negative potential effect of prosthesis characteristics, as previously observed [24]. Over-contoured restorations, defined as the presence of a proximal emergence angle >30 degrees, were not associated with impaired peri-implant tissue conditions. Previous studies have demonstrated that the emergence profile angle had no impact on tissue-level implant conditions, contrary to with the bone-level implants, suggesting that some implant factors had a compensatory positive effect [18, 22]. Keratinized mucosa with a width ≥2 mm was associated with higher BOPi and PiPD. The impact of keratinized mucosa width on peri-implant tissue health is still debated [1, 5]. Soft tissue thickness measured during implant surgery has been shown to be associated with PiPD, BOPi, and Bli increases during the follow-up in patients with a history of periodontitis [48]. Low keratinized mucosa width has been related to plaque retention, inflammation, and increases in recession [53, 54], but the levels of BOPi and PiPD were considerably higher compared with those in the present study. Furthermore, the low soft tissue retraction around implant compared to teeth may have been responsible for the higher measured value of PiPD. These results showed that implant and prosthesis characteristics mainly influenced plaque, soft and bone tissue levels around implants, and could modify the impact of periodontal parameters on peri-implant status.

Multivariable regression analyses of the associations between peri-implant status and demographic, periodontal, implant, and prosthesis parameters confirmed that the peri-implant conditions were still influenced by the periodontal patient conditions regardless of other parameters. The present study identified different profiles of risk indicators that depended on peri-implant parameters. The mean CALi and IPS were influenced by the mean CAL and FMPS, respectively, and not by demographic, implant, and prosthesis characteristics, confirming the predominance of the patient periodontal environment on-site risk indicators. The mean PiPD and BOPi were associated with the corresponding periodontal parameters, the mean PPD and BOP, but appeared also influenced by other site factors, such as prosthesis factors for the mean PiPD and patient factors, such as smoking for the BOPi. Using multivariable regression analysis, a strong relationship between Bli and BL/age appeared, while Bli associations with implant factors, i.e., location, implant type, and implant number, were still observed. Such types of independent associations have been previously described for the mean PPD and CAL [31]. In other cross-sectional studies using a multivariable analysis approach to investigate the final impact of various patient and site factors on peri-implant status/disease occurrence, the independent association between periodontal patient and implant condition was not systematically observed [20, 21, 24, 32, 33, 55]. For instance, in the study of Kissa et al. [20], the impact of periodontitis history on PiPD was not observed after multivariable analysis. The same effect was seen in the study of Pimentel et al. [32] for the impact of PPD ≥6 mm on peri-implantitis. In the study of Dalago et al. [21], a history of periodontitis appeared to be a risk indicator of peri-implantitis only after multivariable analysis was conducted with an OR = 2.2. These different observed associations may reflect the variability of clinical parameter interactions in the studied populations, as well as the use of different peri-implant disease definitions [47]. In the present study, peri-implant soft-tissue diseased sites defined as PiPD > 4 mm + BOPi remained independently associated to some demographic, periodontal, implant, and prosthesis parameters, suggesting that these parameters could influence peri-implantitis triggering, as shown previously for other periodontal parameters in a similar cohort [28].

The absence of data on peri-implant tissue conditions after healing may be a limitation in interpreting a cause-and-effect relationship between the patient/site parameters. The percentages/numbers of peri-implantitis and diseased sites with PiPD ≥6 mm and BOPi were very low and did not allow association analyses. However, the purpose of this work was first to compare the implant and periodontal status and their associations during implant follow-up. The one-year function was chosen as the minimal follow-up duration requirement for patient inclusion because it allowed a sufficient time for soft peri-implant tissue and bone initial adaptation to the oral environment and for their stabilization to occur [16, 56]. Furthermore, this time was frequently chosen in comparable studies [20, 21, 24, 32, 43]. There were some characteristics that could have influenced data recording, such as over-contoured prostheses, and may have affected the accuracy of IPS and probing depth assessments [15]. Other potential specific implant site factors described in the literature were not considered here, such as the implant surgical procedures [17, 44] and compliance [33, 57]. The limited final number of included patients as well as the specificity of the studied population suggested that results could not be directly generalized to all patients treated in the Department of Periodontology at Strasbourg and other populations. However, the ratio between finally included and eligible patients (51%) was similar or higher than the ratios observed in other comparable studies, 50.2% [42], 44.4% [33], and 39.8% [32].

## 5. Conclusions

The present study demonstrated that peri-implant and periodontal soft tissue statuses were different as a potential consequence of the initial implant and prosthesis procedure healing. However, during the implant follow-up, peri-implant parameters were predominantly associated with their corresponding periodontal parameters regardless of their associations with the implant and prosthesis characteristics. These results demonstrated that in addition to the pathologic link between periodontal and peri-implant diseases, a clinical association between the periodontal and peri-implant soft tissue conditions could be also observed during follow-up procedures.

## Figures and Tables

**Table 1:** Patient periodontal and peri-implant characteristics.

Characteristics	Teeth	Implants
*Diagnosis and treatment*		
Periodontitis stage I/peri-implant health nb (%)	2 (1.86)	14 (13.08)
Periodontitis stage II/mucositis nb (%)	38 (35.51)	90 (86.53)
Periodontitis stages III–IV/peri-implantitis nb (%)	67 (62.62)	3 (2.8)
Follow-up/mean time in function years (SD)	12.82 (6.71)	7.22 (3.66)
Nb teeth/Nb implants (SD)	21.91 (5.26)	2.95 (2.2)
% FMPS/IPS (SD)	**23.64 (17.05)**	16.5 (23.37)

*Periodontal and peri-implant tissues*		
% BOP/BOPi (SD)	14.4 (11.43)	**25.91 (20.8)**
Mean PPD/PiPD mm (SD)	2.4 (0.39)	**2.72 (0.6)**
% PPD/PiPD >4 mm + BOP/BOPi (SD)	1.58 (3.17)	**4.77 (9.73)**
Mean CAL/CALi mm (SD)	**3.29 (88.35)**	2.73 (88.73)
% REC/RECi >1 mm (SD)	**27.59 (22.17)**	4.21 (9.82)
BL/age/Bli mm (SD)	0.58 (0.23)	0.86 (0.71)

FMPS: full mouth plaque score, IPS: implant plaque score, BOP/BOPi: bleeding on probing on teeth/implants, PPD/PiPD: pocket probing depth on teeth/implants, CAL/CALi: clinical attachment level on teeth/implants, REC/RECi: gingival/mucosa recession on teeth/implant, BL/age, % of bone loss of the most affected tooth divided by patient age, Bli: mean bone loss of the most affected sites per implant, SD: standard deviation. Nb: number. In bold *P* < 0.05 for comparisons between soft periodontal and peri-implant tissue characteristics using the Wilcoxon test.

**Table 2:** Univariable regression analysis of associations between the demographic, periodontal, and peri-implant tissue characteristics at the implant level.

Characteristics	Mean PiPD			Mean CALi			Bli			IPS			BOPi			% PiPD >4 mm + BOPi		
	Value	*P*	CI (95%)	Value	*P*	CI (95%)	Value	*P*	CI (95%)	Value	*P*	CI (95%)	Value	*P*	CI (95%)	Value	*P*	CI (95%)
Age	0.001	0.886	(−0.011, 0.013)	−0.001	0.881	(−0.016, 0.014)	0.001	0.947	(−0.016, 0.017)	0.003	0.221	(−0.002, 0.007)	−0.005	**0.009**	(−0.008, −0.001)	−0.001	0.492	(−0.003, 0.001)
Gender women	−0.111	0.289	(−0.316, 0.094)	−0.117	0.348	(−0.362, 0.128)	−0.136	0.339	(−0.414, 0.143)	−0.014	0.686	(−0.083, 0.055)	0.030	0.339	(−0.031, 0.091)	0.040	**0.017**	(0.008, 0.072)
Nonsmoker/smoker	−0.202	0.228	(−0.529, 0.125)	−0.134	0.506	(−0.527, 0.26)	−0.219	0.328	(−0.657, 0.219)	0.014	0.806	(−0.098, 0.127)	0.070	0.167	(−0.029, 0.168)	0.007	0.779	(−0.045, 0.06)
Ex-smoker/smoker	−0.118	0.490	(−0.451, 0.216)	−0.055	0.789	(−0.457, 0.348)	0.088	0.697	(−0.357, 0.534)	0.020	0.740	(−0.096, 0.135)	0.037	0.472	(−0.063, 0.137)	−0.008	0.763	(−0.061, 0.045)
Nb implants	0.029	0.155	(−0.011, 0.069)	0.056	**0.026**	(0.008, 0.104)	0.089	**0.001**	(0.04, 0.137)	−0.001	0.876	(−0.016, 0.013)	−0.015	**0.014**	(−0.026, −0.004)	−0.001	0.824	(−0.007, 0.006)
FMPS	0.007	**0.004**	(0.002, 0.012)	0.012	**<0.001**	(0.007, 0.018)	0.007	0.059	(0, 0.013)	0.005	**<0.001**	(0.004, 0.006)	−0.001	0.069	(−0.003, 0)	0.000	0.539	(−0.001, 0.001)
BOP	0.002	0.556	(−0.006, 0.01)	0.002	0.668	(−0.007, 0.011)	0.001	0.922	(−0.01, 0.012)	0.001	0.242	(−0.001, 0.004)	0.004	**0.002**	(0.001, 0.006)	0.001	0.318	(−0.001, 0.002)
Mean PPD	0.569	**<0.001**	(0.322, 0.816)	0.649	**<0.001**	(0.35, 0.948)	0.279	0.139	(−0.09, 0.648)	0.132	**0.004**	(0.045, 0.22)	−0.015	0.724	(−0.097, 0.067)	0.012	0.593	(−0.031, 0.055)
%PPD>4 mm + BOP	4.787	**0.003**	(1.736, 7.838)	5.167	**0.010**	(1.287, 9.047)	1.688	0.447	(−2.658, 6.034)	0.618	0.271	(−0.48, 1.717)	0.363	0.463	(−0.605, 1.332)	0.666	**0.008**	(0.194, 1.137)
Mean CAL	0.299	**<0.001**	(0.217, 0.382)	0.519	**≤0.001**	(0.381, 0.656)	0.049	0.474	(−0.086, 0.185)	0.034	**0.024**	(0.005, 0.063)	0.004	0.811	(−0.026, 0.033)	0.030	**0.001**	(0.013, 0.046)
BL/age	0.081	0.723	(−0.369, 0.532)	0.306	0.263	(−0.229, 0.84)	0.460	0.134	(−0.139, 1.059)	0.060	0.440	(−0.092, 0.213)	−0.010	0.886	(−0.146, 0.126)	−0.008	0.829	(−0.077, 0.062)

Ex-smoker: former smoker; FMPS: full mouth plaque score; IPS: implant plaque score; PPD/PiPD: pocket probing depth on teeth/implant; CAL/CALi: clinical attachment level on teeth/implants; BOP/BOPi: bleeding on probing on teeth/implant; BL/age: % of bone loss of the most affected tooth divided by patient age; BLi: bone loss of the most affected implant site; Nb: number, CI: confidence interval. In bold, *P* < 0.05.

**Table 3:** Implant and prosthesis characteristics.

*Peri-implantitis characteristics*	
Nb implants without peri-implantitis (%)	307 (99.04)
Nb implant with peri-implantitis (%)	3 (0.96)

*Implant characteristics*	
Time in function years (SD)	7.73 (4.34)

*Location*	
Anterior (canine, incisor) nb (%)	55 (17.7)
Posterior maxilla (premolar, molar) nb (%)	126 (40.65)
Posterior mandible (premolar, molar) nb (%)	129 (41.61)
Tissue-level implant nb (%)	295 (95.16)

*Prosthesis characteristics*	
Keratinized mucosa width ≥2 mm nb (%)	228 (73.55)
Cemented prosthesis nb (%)	280 (91.21)
Emergence profile angle >30 degrees nb (%)	118 (38.06)
Bridge restoration support nb (%)	118 (38.06)
Misfitting nb (%)	61 (19.68)

**Table 4:** Univariable regression analysis of associations between the implant, prosthesis, and peri-implant tissue characteristics at the implant level.

Characteristics	Mean PiPD			Mean CALi			BLi			IPS			BOPi			% PiPD >4 mm + BOPi		
	Value	*P*	CI (95%)	Value	*P*	CI (95%)	Value	*P*	CI (95%)	Value	*P*	CI (95%)	Value	*P*	CI (95%)	Value	*P*	CI (95%)
*Implant characteristics*																		
Time in function	−0.005	0.696	(−0.028, 0.018)	0.005	0.707	(−0.022, 0.032)	0.014	0.399	(−0.018, 0.045)	0.000	0.922	(−0.007, 0.006)	−0.009	**0.009**	(−0.016, −0.002)	−0.001	0.574	(−0.005, 0.003)
Postmand/Postmax	−0.279	**0.002**	(−0.457, −0.101)	−0.194	0.061	(−0.396, 0.009)	−0.199	0.127	(−0.454, 0.057)	0.029	0.228	(−0.018, 0.077)	−0.001	0.981	(−0.056, 0.054)	−0.041	**0.012**	(−0.073, −0.009)
Anterior/Postmax	−0.022	0.848	(−0.251, 0.206)	0.003	0.980	(−0.255, 0.262)	0.403	**0.016**	(0.075, 0.732)	0.000	0.995	(−0.06, 0.06)	−0.038	0.287	(−0.109, 0.032)	−0.024	0.248	(−0.066, 0.017)
Bone-level implant	−0.254	0.281	(−0.717, 0.208)	−0.219	0.426	(−0.759, 0.321)	1.178	**≤0.001**	(0.569, 1.788)	−0.069	0.317	(−0.206, 0.067)	−0.000	0.994	(−0.141, 0.14)	0.001	0.988	(−0.074, 0.075)

*Prosthesis characteristics*																		
KMW≥ 2 mm	0.204	**0.026**	(0.025, 0.384)	0.014	0.894	(−0.188, 0.215)	−0.034	0.798	(−0.297, 0.228)	−0.024	0.301	(−0.07, 0.022)	0.064	**0.022**	(0.009, 0.118)	0.024	0.146	(−0.008, 0.056)
Cemented prosthesis	0.324	**0.051**	(0, 0.649)	0.498	**0.009**	(0.125, 0.871)	−0.300	0.193	(−0.752, 0.152)	0.073	0.130	(−0.022, 0.168)	−0.021	0.679	(−0.118, 0.077)	0.045	0.106	(−0.009, 0.099)
EP angle >30°	−0.243	**0.003**	(−0.406, −0.081)	−0.197	**0.035**	(−0.379, −0.014)	0.131	0.281	(−0.108, 0.37)	−0.002	0.927	(−0.044, 0.04)	−0.014	0.575	(−0.064, 0.036)	−0.016	0.283	(−0.046, 0.013)
Bridge restoration	0.050	0.609	(−0.142, 0.243)	0.044	0.698	(−0.179, 0.266)	0.364	**0.007**	(0.101, 0.627)	0.018	0.534	(−0.038, 0.074)	−0.079	**0.008**	(−0.136, −0.021)	0.008	0.634	(−0.025, 0.041)
Misfitting nb (%)	0.222	**0.034**	(0.017, 0.428)	0.154	0.190	(−0.077, 0.386)	−0.080	0.600	(−0.379, 0.219)	−0.049	**0.020**	(−0.09, −0.008)	−0.045	0.163	(−0.107, 0.018)	−0.011	0.528	(−0.047, 0.024)

PiPD: implant pocket probing depth; CALi: implant clinical attachment level; BLi: bone loss of the most affected implant site; IPS: implant plaque score; BOPi: implant bleeding on probing. Anterior: anterior location; Postmand: posterior mandible location; Postmax: posterior maxilla location; KMW: keratinized mucosa width' EP: emergence profile. CI: confidence interval. In bold, *P* < 0.05.

**Table 5:** Multivariable regression analysis of associations between the periodontal, implant, prosthesis, and peri-implant tissue characteristics at the implant level.

Characteristics	Value	*P*	CI (95%)	Value	*P*	CI (95%)	Value	*P*	CI (95%)
	Mean PiPD			Mean CALi			BLi		
Mean PPD	0.548	<0.001	(0.306, 0.79)						
Mean CAL				0.412	<0.001	(0.318, 0.505)			
BL/age							0.797	0.002	(0.302, 1.293)
Anterior/Postmax							0.066	0.004	(0.024, 0.107)
Bone-level implant							1.006	0.001	(0.432, 1.581)
Cemented prosthesis	0.319	0.039	(0.017, 0.621)						
EP angle >30°	−0.186	0.025	(−0.348, −0.024)						
Nb implants							0.066	0.004	(0.024, 0.107)

	IPS			BOPi			% PiPD >4 mm + BOPi		
Gender women							0.038	0.014	(0.008, 0.068)
FMPS	−1.006	0.001	(−1.581, 0.432)						
BOP				0.003	0.011	(0.001, 0.005)			
% PPD >4 mm + BOP							0.611	0.011	(0.157, 1.066)
Nonsmoker/smoker				0.110	0.020	(0.02, 0.2)			
Postmand/Postmax							−0.039	0.016	(−0.071, −0.008)
KMW ≥2 mm				0.058	0.033	(0.005, 0.112)			
Cemented prosthesis							0.059	0.025	(0.008, 0.11)

FMPS: full mouth plaque score, IPS: implant plaque score, BOP/BOPi: bleeding on probing on teeth/implants, PPD/PiPD: pocket probing depth on teeth/implants, CAL/CALi: clinical attachment loss on teeth/implants, BL/age, % of bone loss of the most affected tooth divided by patient age, BLi: bone loss of the most affected implant site. Anterior: anterior location, Postmand: Posterior mandible location, postmax: Posterior maxilla location, KMW: keratinized mucosa width, EP: emergence profile. Nb: number. CI: confidence interval.

## Data Availability

Data available on request from the authors.

## Additional Points

What is known on the topic: (i) peri-implant tissue conditions are influenced by the periodontal, implant, and prosthesis characteristics. (ii) The clinical definitions and assessment tools of peri-implant tissue morbidity need to be better evaluated according to the normal and pathologic implant environments. What the submitted study adds: (i) this study specifies the association of patient and implant profiles with the peri-implant tissue characteristics. (ii) In patients that require periodontal and implant maintenance, the periodontal conditions appear predominantly associated with the peri-implant tissue response to their environment. It is important for clinicians to assess the periodontal treatment outcomes that could reduce peri-implant morbidity.
